# Response to Zamani et al. (2020): The omission of critical data in the pursuit of “revolutionary” methods to accelerate the description of species

**DOI:** 10.3897/zookeys.1033.66186

**Published:** 2021-04-22

**Authors:** Michael Sharkey, Brian Brown, Austin Baker, Marko Mutanen

**Affiliations:** 1 The Hymenoptera Institute, 116 Franklin Ave., Redlands, CA 92373, USA The Hymenoptera Institute Redlands United States of America; 2 Entomology Section, Natural History Museum of Los Angeles County, 900 Exposition Boulevard, Los Angeles, CA 90007, USA Natural History Museum of Los Angeles County Los Angeles United States of America; 3 Department of Entomology, University of California, Riverside, CA, USA University of California Riverside United States of America; 4 Ecology and Genetics Research Unit, University of Oulu, Finland University of Oulu Oulu Finland

## Abstract

Here we respond to the criticisms leveled against a proposal that suggested an efficient solution to the taxonomic impediment. We clarify some of our objectives and demonstrate that many of the criticisms apply more to traditional approaches to taxonomy rather than to our minimalist approach.

## Introduction

Zamani et al. (2020) criticized a solution to the taxonomic impediment proposed in Meierotto et al. (2019), who employed COI barcodes plus a single photograph as diagnostics. The authors of this rebuttal are in full agreement that a diagnostic method similar to the Meierotto et al. and the follow-up Sharkey et al. (2021) approach is needed in groups with overwhelming diversity and little likelihood of ever being treated in a morphological revision. Examples of such groups are the phorid fly genus *Megaselia* Rondani, which is estimated to have close to 1000 species in northeastern Costa Rica alone, and many genera of Neotropical Braconidae, some of which were the subjects of the Meierotto et al. and Sharkey et al. papers. Although the Zamani et al. critique touched on many subjects with which we disagree, we limit our response to what we see as key issues that were not comprehensively addressed in Sharkey et al. (2021).

Much of what Zamani et al. demanded as ***required*** taxonomic procedure is opinion. Taxonomy can proceed in different ways, with different phases of completeness. Detailed treatment of all life stages of an insect, for instance, frequently postdates the description of that species, which is often based on a single sex. Aspects of variation, distribution, life history, etc. are often included later, long after the species is described. Meierotto et al. (2019) took this concept one step further. Using DNA barcoding, which usually allows for a more precise recognition of species, they proposed to defer almost all aspects of species description other than a diagnosis based on COI gene sequence and a photograph. Future revisers may include these omitted details by examining specimens in greater depth, should there be the desire and finances to do so.

The first and perhaps most emphasized criticism of their article was that the, “authors failed to diagnose their 15 new *Zelomorpha* Ashmead, 1900 species from 51 out of 52 previously known species”. An important note is that the molecular diagnoses employed are recognized as valid diagnoses according to the code of zoological nomenclature. In Meierotto et al. it was emphasized that the second author had seen all of the types and that none but *Z. arizonensis* were, in his opinion, conspecific with described species; however, let’s imagine that this was not true and that Meierotto et al. ignored most of the previously described species; i.e., those lacking COI barcode sequences, essentially all but one. The COI barcode is very effective in diagnosing species of *Zelomorpha* and if a previously described species of *Zelomorpha* was barcoded and found to be very (very) similar or identical to one that Meierotto et al. described, their new species hypotheses would be falsified (or reasonably so). That is an effective diagnostic. The type specimens need not be barcoded. Just as in the case of *Zelomorpha arizonensis*, a specimen fitting the description and locality of the type could be barcoded as a proxy. In many groups of organisms in which the majority of the species are described this would not be a viable alternative, however in hyper-diverse groups with only a small percentage of the fauna described the problems created are few and they are far outweighed by the advantages.

Here are the reasons for temporarily ignoring the previously described species and producing barcode-only diagnoses. 1. It allows for the efficient and quick diagnosis of species. 2. No one uses morphological keys and diagnoses to these hyper-diverse taxa, because they do not work. 3. The only way to even suspect that a newly discovered specimen belongs to a morphologically described species is to borrow all related types and visit museums, mostly scattered across Europe and North America. 4. There are so many species in these groups that a morphological key to the small percentage of described forms has little value.

The first reason is explained elsewhere in this article and in Sharkey et al. (2021) so we begin with the second reason, i.e., no one uses the keys and diagnoses in these hyper-diverse groups. There are very few revisionary studies on species-rich genera of braconids in the tropics, but here we will look at statistics for the revision of *Alabagrus* Enderlein, 1920 by Sharkey (1988).

The revision of *Alabagrus* included 104 species treated with morphology only. It contained a key as well as diagnoses and descriptions of each species and was heavily critiqued by Sharkey et al. (2021). According to a search in Google Scholar, the *Alabagrus* revision has 32 citations. The majority of the citations are surveys that simply copy the distributional records that are in the paper. For example, Coronado-Blanco et al. (2016) surveyed the literature for all Agathidinae occurring in Mexico and included the number of species cited as being present in Mexico by Sharkey (1988); the keys and descriptions were not employed.

There are about four citations for the *Alabagrus* revision in which the publication was used to identify a specimen, but in only one of these was the identification verified by anyone other than Sharkey. In this sole citation, a parasitoid of a new Nearctic species of Crambidae, *Diatraea mitteri*, was identified by as *Alabagrus imitatus* (Solis et al. 2015). The key has not been used for any of the Neotropical species where 98% of the diversity lies.

It took Sharkey over seven years and a prolonged trip to Europe to view types to produce the revision and it is worse than useless, because it is full of misleading information on species limits and species distributions. Some might argue for an integrative approach, such as the revision of Costa Rican *Alabagrus* by Sharkey et al. (2018), but what is the point of including morphological descriptions and keys when the COI barcode is the only reliable source for identification? There appears to be good reason to ban revisions based solely on morphology rather than those based primarily on COI barcodes.

The third reason, to temporarily ignore old type specimens until they, or proxies, are barcoded, is the expense and difficulty of viewing them. The only way to even suspect that a newly discovered specimen belongs to a described species is to borrow all relevant types and probably visit museums mostly scattered across Europe and North America. The first problem that a reviser would come across for a revision of *Zelomorpha* would be to find the types of the 52 species of *Zelomorpha* that had been described. Since Sharkey has already done this we can report that it is not possible to find these in the literature. Species of *Zelomorpha* have been described under at least 13 different generic names, i.e., *Agathis*, *Biroia*, *Bracon*, *Chromomicrodus*, *Coccygidium*, *Crassomicrodus*, *Cremnops*, *Dichelosus*, *Disophrys*, *Ichneumon*, *Microdus*, *Spilomicrodus*, and *Zelomorpha*. Most of these are species described by taxonomists in the late 19^th^ and early 20^th^ centuries, therefore the descriptions are brief and all but useless. A reviser would have to visit 12 different museums in 10 different countries [Poland, Hungary, France, Germany, USA (Washington D.C.), USA (Philadelphia), USA (New York), England, Sweden, Costa Rica, Denmark, and Italy] and look at all of their agathidines to “rediscover” the 52 species. After looking at these species there would be many that certainly are not among the species being revised and these could be maintained. The problem occurs when a museum specimen is a close match with a specimen in hand. If not identical and from the same locality as the holotype, morphological similarity is not enough to indicate conspecificity. The article by Sharkey et al. (2021) clearly documents this, as does the revision by Sharkey et al. (2018) in which even with barcodes some species could not be differentiated morphologically. Another example, and there are thousands, is in the phorid genus *Megaselia*, in which 16 species were masquerading as one in collections, until molecular data were collected (Brown et al. in review). In hindsight, a few of these can be identified using morphological characters, but most cannot, even though they have deep COI divergences.

Morphological diagnoses necessitate the viewing of holotypes and as such they act as an enormous impediment to the taxonomy of hyper-diverse taxa. There are also social and environmental issues to consider. The cost of travelling to the museums to view types is expensive, all but precluding the participation of taxonomists from developing countries. There is a cost to the environment in the air travel involved, and finally in the case of pandemics, such as the one currently being experienced (Covid-19), virtually no one can visit museums. Consider the alternative when the barcode serves as a proxy to the holotype; a simple search on BOLD (Barcode of Life Datasystem) will indicate with a great degree of certainty whether a specimen belongs to a described species.

The final argument, for postponing the inclusion of non-barcoded species in our species-rich genera, is the low probability that newly discovered specimens have been described. We estimate that there are approximately 500 species of *Zelomorpha*. With only 52 described there is only a 10% chance that a newly described species will be a synonym. Compare this to the current 33% synonymy rate for species of Ichneumonoidea as documented by Yu et al. (2016). We can also imagine a day in which all 500 species of *Zelomorpha* are described and there is a morphological key to them all. Such a key, if dichotomous, would be about 700 couplets long. Anyone familiar with long keys will know that the longer the key is, the higher the probability of error is. Multiple entry keys such as Delta and Lucid can reduce the decisions required for identification but the probability of success would still be minimal. In summary we feel that little is lost if types are ignored until they or their proxies are barcoded, and we emphasize that barcoding types should be a priority for museums. We further suggest that it is time to stop describing members of these species-rich genera based on morphology alone.

Zamani et al.’s statements that more than one photograph should be required and that text elucidating important diagnostic characters should be included are irrelevant because ***we don’t expect anyone to identify these species using morphology***. For some groups of insects (we are not saying for all groups), the idea that people, even experts, can accurately identify species using morphology is wrong, as documented separately (Sharkey et al. 2021; Brown et al., in review). For instance, hand a *Megaselia* specimen from Costa Rica to a phorid taxonomist, and outside of a couple of well-known species, the possibility of getting an identification back is almost zero. Experts can try to run the specimen through the inadequate keys (that treat only a small fraction of the 2000+ species estimated from Costa Rica), compare it to the broken, shriveled types, and perhaps look through the almost non-existent identified collections to try to get close. But even if a specimen is matched to a description, that specimen might be one of a group of cryptic species that may not be recognizable (using some extremely minute or subtle morphological character) without sequence data. With a likely total of over 2,000 species of Costa Rican *Megaselia*, this situation will not change soon, if ever.

Users of biodiversity information need to be able to recognize species. This will never occur using morphology alone in most species-rich groups of insects with cryptic morphologies. Such organisms require huge amounts of time to diagnose, with required dissections, drawings, and incorporation into ever-longer keys with more and more complicated exceptions. Additionally, such morphological keys need to be generated for all life stages separately, seriously compromising a system based solely on morphology. DNA barcodes have a huge benefit of permitting species delimitation and specimen identification regardless of life history stage, and usually also sample condition.

To us, one of the least-appealing aspects of the realization that barcoding is necessary is the loss of the fantasy that we can sit down with a specimen at a microscope and definitively arrive at a species name for it. This type of immediate identification in some highly diverse groups of insects is a taxonomic fiction. Until we have individual-sized barcoders, which are not far off (Pomerantz et al. 2018), identification in groups, such as Neotropical Braconidae and Phoridae, is an event involving the processing of at least one 96-well plate of specimens. Taxonomists can come close (such as a genus, or species-group identification), but nobody should need to spend 10–30 minutes in a usually futile attempt to identify a specimen when anyone can obtain a conclusive answer with barcodes, 96 at a time. A taxonomist’s time is too valuable for this; instead, they should be overseeing the results of barcoding, looking for errors, split or lumped taxa that occur at extremely low levels in barcoding (much lower levels than with morphological taxonomy, in our experience), not to mention publishing the new species that have been discriminated by COI sequences. Costs for this procedure will decrease dramatically over time, and we have to prepare for this reality now. In fact, some of the newest high-throughput platforms, notably those by Pacific Biosciences, already allow analyses of thousands (SEQUEL I) (Hebert et al. 2018) or tens of thousands (with the newest upgrade of SEQUEL II) of specimens at costs ($1–$2 USD) that are only a fragment of those of the Sanger methods. This unprecedented progress on the DNA sequencing technology front seriously challenges the cost-efficiency of morphology-based description and identification approaches under most circumstances.

One oft-repeated criticism of this approach (in both Zamani et al., and in a barrage of social media posts) is that it discriminates against entomologists in developing countries, where funding for this type of work might not exist. This argument is beside the point for groups like ours that have so many species that morphology simply does not work. It is not DNA taxonomy that is the problem for scientists in developing countries; it is the large groups that cannot be treated in the traditional way. If critics want to argue that it is better to leave these groups “undone” than to treat them in a way that some cannot afford to replicate, we have to respectfully disagree.

Zamani et al.’s comments invite contemplation about the target audience for longer descriptions. Applied users of biodiversity information (conservation biologists, ecologists) don’t need to know how many notopleural setae a fly has; however, they need to identify specimens, know which species are present in a given area, and where else a given species might occur. The exhaustive descriptions of most taxonomists of hyper-diverse groups only serve themselves and a few other taxonomists; in fact, we venture a bet that few have used the species-level keys in major taxonomic revisions of hyper-diverse genera of Ichneumonoidea, Phoridae and many others. Despite the fact that there are tens of thousands of species in these groups, there are very few major revisions.

Meanwhile, as we generate time-consuming morphological treatments of a very small percentage of our faunas, global warming is on the rise, wildfires are burning at record rates, the loss of natural habitats is accelerating, and thousands of species are going extinct. It is important to note that the small fraction of all species so far described largely represent the less diverse groups with large body size, and that this work has mostly been done in the least biodiverse areas such as Europe. It is, therefore, foreseeable that taxonomic work, if continuing to rely largely on morphology, will progress even more slowly and become increasingly complex than during the past ~260 years. We do not have time to wait but must find novel and better solutions for the taxonomic crisis.

Stating that our form of description, as a first-pass step for taxonomy, is unacceptable, sloppy, or lazy is untrue; it is simply efficient towards a different goal. A DNA-based taxonomy will quickly make species known in large numbers that otherwise would remain in obscurity. The critics’ concern about description quality seems to be conflating comprehensiveness of descriptions (how many characters are mentioned or illustrated) with accuracy of the descriptions. We are concerned with accurately and concisely describing new species. Therefore, possible objections to DNA-based species are that they aren’t real species and that the species we describe cannot be recognized later by other researchers.

The reality of species could be argued ad nauseam depending on one’s preferred species concept. As speciation is a process rather than an event, delimitation of species is also inherently subjective, for example with allopatric populations slowly diverging apart (Mutanen et al. 2012). We argue that DNA-based species as identified by BINs in BOLD are highly objective and congruent with species identified using multiple genes more than 90% of the time for our taxa, phorid flies and braconid wasps. For the latter, the success rate is 98% (Sharkey et al. 2021). This experience is at odds with Meier and Zhang (2009), who cite a 34% error rate in a data set from another public database, GenBank, but we suspect that their number is a product of “operational” errors, such as incorrect identifications in GenBank, contaminated sequences, incomplete sequences, and other easily-corrected items that would be amended in any serious taxonomic analysis. Furthermore, such comparisons typically assume the reference taxonomy, usually morphology-based, to be accurate, which hardly ever is true (Mutanen et al. 2016). We find a much lower discrepancy in BOLD, with only a few BINs needing refinement by taxonomists, either by examining the morphology of specimens, ecological factors like host use, or patterns of COI divergence. This experience tells us that intelligent shortcuts are possible in completing the inventory of large groups. Admittedly, study of interesting information about the structure and evolution of these species is deferred until later, but this is in service of the priorities of those needing biodiversity data now (ecologists and conservation biologists), rather than the systematists who want a better understanding of their group.

Another criticism is the poor quality of images. We agree with this concern, which was a mechanical problem in the processing of the article, which has now been resolved for both the Meierotto et al. and Sharkey et al. (2021) publications.

We share the same ideals as Zamani et al. concerning taxonomic treatments, i.e., employing multiple genes to elucidate species boundaries and place species in a phylogenetic context, multiple images of each holotype and other specimens to show variation, an illustrated morphological key, and a concise morphological diagnosis, e.g., Sharkey et al. (2018), Brown (2006), Brown and LeBrun (2010). As clearly documented by Meierotto et al., given the number of undescribed species of Ichneumonoidea and the current rate of species descriptions, it would take thousands of years to treat all Ichneumonoidea with this level of detail. We suggest that the Meierotto et al. approach, or something akin to it, is the most promising proposed solution that can act as a first taxonomic pass and one that can easily be built upon when time, money, and desire permit. Currently, the most productive 10 ichneumonologists each describe approximately 500 species in a lifetime. With the Meierotto et al. approach it will be easy for a productive taxonomist to treat 1000 species each year. For example, in our first attempt at a large species treatment, Sharkey et al. (2021) described more than 400 new species of Costa Rican braconids while trying to streamline the process. Fifty years from now we could have 20,000 ichneumonoid species treated the conservative way or one million using the Meierotto et al. approach (20 taxonomists × 1000 species per year × 50 years). One million species is the current estimate we have for total ichneumonoid species-richness. These two approaches are not mutually exclusive, and the species recognized using the Meierotto et al. approach may drastically increase the rate of more thorough second pass revisions.

A point made by Zamani et al. is that mitochondrial diagnoses are flawed because “*Wolbachia* may be altering mtDNA introgression” and “mitochondrial trees often disagree with nuclear species trees.” This has been shown to be true in some cases (Klopfstein et al. 2016, Ivanov et al. 2018); however, as a first pass, DNA barcoding will still outperform morphology-based species recognition for highly cryptic taxa. Additional splitting of species that are discovered to share COI sequences may be necessary if more genetic data are acquired, but this is no different than any other taxonomic revisionary approach.

Zamani et al. opined that DNA-based descriptions will make the identification of millions of historical specimens impossible. This is only a short-term problem, however, as technology is rapidly improving the sequence capture rate of historical specimens. Once this technological hurdle is passed, collections will be gold mines of information on the historical distributions of species (many of which will presumably be extinct). “Museomics” is indeed a rapidly developing area of taxonomy, including DNA barcoding (Prosser et al. 2016); for example, the Finnish Barcode of Life initiative is presently barcoding old museum specimens, including types, on a large-scale.

Zamani et al. were inaccurate in their statement, “Simply assigning all BINs taxonomic names as Meierotto et al. (2019) propose would indeed complete the inventory of life on Earth extremely quickly”. This is a potential solution; however, Meierotto et al. did not advocate this approach for several reasons. The most relevant is that BINs do not equal species: more than one species may occupy a BIN, and even more rarely a species may occupy more than one BIN. A 2% genetic distance is the conventional threshold for species delimitation using COI barcodes (Jones et al. 2011), and this is used to cluster putative species, but it is not infallible and was never proposed as such (Ratnasingham and Hebert 2013). For example, in their treatment of Costa Rican braconids, Sharkey et al. (2021) found seven species of *Macrocentrus* in one BIN, and this necessitated a morphological key to differentiate them (although their COI sequences also distinguished them but not at a level to allow for separate BIN placements). Another potential problem is contamination in cases where a COI barcode is assigned to the wrong specimen. This also requires an examination of the specimens and some expertise. Finally, it takes a great deal of expertise to identify any specimen in our diverse taxa to the generic level, and this is a necessary first step for any revision. Zamani et al. complained that the approach would supplant taxonomists with technicians. This is neither entirely true nor entirely problematic. Technicians will play an increasingly important role, and many of the co-authors of the Sharkey et al. (2021) treatment of Costa Rican braconids fit the technician category. Enabling technicians to do much of the data acquisition will reciprocally enable taxonomists to focus their time and effort on problems that require their expertise.

Zamani et al. suggest that, “a true paradigm shift in taxonomy will come only when there is a revolution in the level of financial investment in taxonomy”. We have heard this for decades as resources for alpha taxonomy steadily decline. This cry for help is ignored by the general public and by scientists in other disciplines. Many taxonomists fail to realize that 99.99 percent of the public have no idea what we do and could not care less about the description of a new species of *Zelomorpha* or any other insect. Greatly increased funding for alpha taxonomy will never happen unless we taxonomists can demonstrate to funding agencies that we can overcome the taxonomic impediment in a reasonable timeframe with a reasonable budget. This could be done if those of us working on understudied, hyper-diverse taxa employed an approach similar to the one outlined by Meierotto et al. (2019) and Sharkey et al. (2021). Science funders seem to agree with this statement and three examples follow. The International Barcode of Life (iBOL) and its participatory nations raised $125 million for the first phase of BARCODE 500K (https://ibol.org/programs/barcode-500k/). The BioAlfa project, supported by Costa Rican government and others (https://www.gdfcf.org/bioalfa-bioliteracy-costa-rica), has begun to barcode all of Costa Rica’s multicellular terrestrial life-forms over ten years, with millions of dollars in start-up funding and sweat equity. And finally, the European Research Council awarded ~12 million Euros for the global-scale biodiversity initiative LIFEPLAN, with massive DNA barcoding being at its heart (https://www2.helsinki.fi/en/projects/lifeplan).

It is interesting to contemplate the degree of damage that could be done to the taxonomy of a group if the worst nightmares of Zamani et al. were realized and a DNA-based description paradigm was widely adopted. Many species would be described quickly, some incorrectly. Perhaps 1% of descriptions (based on Sharkey’s observations in Braconidae; perhaps more in some other groups) would be wrong or need further fine-tuning. Compare this to the 33% synonymy rate for ichneumonoids that can be extrapolated from Taxapad (Yu et al. 2016). In the last update of this database in 2016, there were 44,385 valid species names and 13,606 synonyms. This 33% synonymy rate does not include lumped species, i.e., species concepts that contain more than one species, which is an even more prevalent source of error. Meanwhile, thousands of new species would be known that would have remained in obscurity. There would be photographs, the means to recognize them based on barcodes, and type material deposited in museums. It is an imperfect system, but it leads to progress on groups that will otherwise remain untouched for decades or perhaps forever. Who, for example, is going to describe the estimated 1,800,000 species of gall midges (Cecidomyiidae) (Hebert et al. 2016)? The answer is obvious; nobody will do it unless new, fast, efficient methods are employed.

For those readers that see the rationale in the above arguments, the question becomes how to effect this change. To implement a survey of megadiverse taxa, it is financially, and in many other respects, impractical to attempt to survey the entire world now. However, all long journeys begin with a few tentative steps, and adoption of DNA-based methods for sorting large collections of specimens is a positive development that will move us closer to this goal.
